# Cloning and Biochemical Characterization of a Hyaluronate Lyase from *Bacillus* sp. CQMU-D

**DOI:** 10.4014/jmb.2209.09036

**Published:** 2022-12-07

**Authors:** Lu Wang, Qianqian Liu, Xue Gong, Wenwen Jian, Yihong Cui, Qianying Jia, Jibei Zhang, Yi Zhang, Yanan Guo, He Lu, Zeng Tu

**Affiliations:** 1 Department of Pathogen Biology, College of Basic Medical Science, Chongqing Medical University, Chongqing, 400016, P.R. China; 2 Department of Infectious Diseases, The First Affiliated Hospital of Chongqing Medical University, Chongqing 400016, P.R. China; 3 International Medical College, Chongqing Medical University, Chongqing, 400016, P.R. China

**Keywords:** Hyaluronate lyase, hyaluronic acid, *Bacillus*, characterization

## Abstract

Hyaluronidase (HAase) can enhance drug diffusion and dissipate edema by degrading hyaluronic acid (HA) in the extracellular matrix into unsaturated HA oligosaccharides in mammalian tissues. Microorganisms are recognized as valuable sources of HAase. In this study, a new hyaluronate lyase (HAaseD) from *Bacillus* sp. CQMU-D was expressed in *Escherichia coli* BL21, purified, and characterized. The results showed that HAaseD belonged to the polysaccharide lyase (PL) 8 family and had a molecular weight of 123 kDa. HAaseD could degrade chondroitin sulfate (CS) -A, CS-B, CS-C, and HA, with the highest activity toward HA. The optimum temperature and pH value of HAaseD were 40°C and 7.0, respectively. In addition, HAaseD retained stability in an alkaline environment and displayed higher activity with appropriate concentrations of metal ions. Moreover, HAaseD was an endolytic hyaluronate lyase that could degrade HA to produce unsaturated HA oligosaccharides. Together, our findings indicate that HAaseD from *Bacillus* sp. CQMU-D is a new hyaluronate lyase and with excellent potential for application in industrial production.

## Introduction

Hyaluronic acid (HA) is a high molecular weight glycosaminoglycan distributed in the extracellular matrix (ECM) of mammalian connective tissues and tumor tissues [1]. HA consists of β-1, 3-N-acetyl-D-glucosamine, and D-glucuronic acid disaccharide units [2, 3], and plays a vital role in water retention, extracellular space maintenance and osmotic pressure regulation of all the membranes [4]. In addition, HA is involved in cell proliferation and migration, wound healing, inflammation, and angiogenesis [5] and is now widely used in medicine, cosmetics, and pharmaceutical fields [6].

Hyaluronidases (HAases) are a group of enzymes that can degrade HA into low- molecular- weight, unsaturated HA oligosaccharides [7]. These products could accelerate the proliferation and differentiation of endothelial cells and regulate the invasive behavior of tumor cells [8, 9]. In addition, local HAase injection could promote the diffusion of drugs and dissipate edema, making it useful in anesthesiology and medical cosmetology [10, 11].

Microbial HAases are recognized as good sources for HAase development with potential clinical applications. They are found in *Bacillus* [12, 13], *Streptococcus* [14, 15], *Streptomyces* [16], and other bacteria strains [17-19]. Most microbial HAases are hyaluronate lyases, which are convenient for operation and cost-effective for fermentation [17]. Nowadays, hyaluronate lyases are being widely used for the preparation of oligo-HA and low molecular HA [20]. As such, better microbial HAases are in high demand.

In our previous study, a *Bacillus* sp. CQMU-D strain that was screened and selected from deep soil in Chongqing, China, was shown as having the potential to produce novel HAase (HAaseD) [21]. Here, we further investigated the cloning, expression, and enzymatic properties of recombinant HAaseD, and our findings should provide new insights into HAaseD and its potential applications.

## Materials and Methods

### Materials

Sodium hyaluronate was purchased from Bloomage Biotech. (China). Chondroitin sulfate A (CS-A) from bovine cartilage, chondroitin sulfate B (CS-B) from pig cartilage, and chondroitin sulfate D (CS-D) from chicken cartilage were obtained from Biomei Biotech Co., Ltd. (China). Meanwhile, the restriction endonuclease was purchased from Thermo Fisher Scientific (USA), and the T4 DNA ligase from Takara (China).

### Sequence Analysis of Gene *hysA*

The protein molecular weight (MW) and isoelectric point (pI) were estimated using the Protparam tool of the Expasy web server (http://expasy.org). The signal peptide sequence of the protein was predicted using the online tool SignalP 5.0 server (http://www.cbs.dtu.dk/services/SignalP). Conserved Domain (CD) Search Service was used to identify protein modules and domains. The protein sequence similarity search was conducted using the BLASTP algorithm on NCBI (https://blast.ncbi.nlm.nih.gov). The phylogenetic tree was constructed with MEGA-X using the NJ algorithm[22].

### Homology Modeling and Molecular Docking of HAaseD

The SWISS-MODEL (https://swissmodel.expasy.org) server was used to assess the protein structure using xanthan lyase (SMTL ID: 2e24. 1) from *Bacillus* sp. as the template. The structure was superimposed and described by PyMOL. To better understand the interactions between HAaseD and model substrate HA, the chemical structure data of substrate HA was downloaded from the PubChem database (https://pubchem.ncbi.nlm.nih.gov/), and imported into ChemBio3D Ultra energy minimum 14.0. The optimized small molecules were imported into AutodockTools for hydrogenation, charge calculation, charge distribution, and rotatable bond setting. PyMOL and Discovery Studio 2019 were used to analyze the interaction mode of the docking results. [23].

### Cloning, Expression, and Purification of Recombinant HAaseD

The full-length gene *hysA* was cloned using forward primer F (5’-CGGGATCCGATGATACTGCAAATCGTTTG-3’) and reverse primer R (5’-CCCTCGAGTCTAATCGAATGTGGACTGTT-3’), and the restriction sites are underlined. HAaseD gene was digested by BamH I and Xho I restriction endonuclease and ligated with pET-32a (+) plasmid for transformation into *E. coli* BL21 (DE3) competent cells. The expression of the recombinant strain was induced at OD_600_, reaching 0.6 with 0.2 mM IPTG for 24 h at 25°C.

Supernatants were obtained by ultrasonic crushing and centrifugation, and the protein was purified with His-tag purification resin by using AKTA purifiers (USA). The protein was eluted by a phosphate buffer containing 100 mM imidazole at a flow rate of 0.5 ml/min. The purity and MW of HAaseD were analyzed using sodium dodecyl sulfate-polyacrylamide gel electrophoresis (SDS-PAGE) on a 10% (w/v) resolving gel. Protein concentration was measured using the BCA protein assay kit.

### Enzymatic Activity Assay

A 100 μl enzyme sample (10 μg/ml) was added to 900 μl 0.2% (w/v) HA substrate solution at 40°C for 10 min. Ultraviolet (UV) absorption of the reaction solution was then measured at 232 nm using a UV2600 Spectrophotometer (Japan), and the same corresponding inactivated enzyme solution was added as a blank. One unit (U) of hyaluronate lyase activity was defined as the amount of enzyme required to release 1 μmol of the unsaturated hyaluronate disaccharide using the millimolar absorption coefficient value of 5,500 M^-1^ cm/min [24].

### Characterization of HAaseD

To determine the optimum temperature, HAaseD (10 μg/ml) and 0.2% (w/v) HA were incubated at 20-80°C for 30 min. HAaseD was incubated at 20-80°C for 1 h, and the residual activity was measured at 40°C for 30 min to determine the thermal stability of HAaseD. Then, HAaseD was reacted with HA substrate in different buffers at 40°C to determine the optimum pH, including citric acid-Na_2_HPO_4_ (pH 2-5), Na_2_HPO_4_-NaH_2_PO_4_ (pH 6-8), glycine-NaOH (pH 9-10), and Na_2_HPO_4_-NaOH (pH 11-12). To determine the effect of pH value on enzyme stability, HAaseD was incubated in different buffers for 2 h at 4°C, and the residual activity was measured at 40°C for 30 min. The effects of metal ions on HAaseD activity were investigated using NaCl, KCl, LiCl, MgCl_2_, CaCl_2_, NiCl_2_, and BaCl_2_ at concentrations of 10, 50, and 100 mM. Similarly, the effects of chelators (EDTA) and surfactants (SDS) on the activity of HAaseD were investigated. Meanwhile, the degradation preference of HAaseD to different substrates was determined using several different substrates (HA, CS-A, CS-B, and CS-D), 100 μl HAaseD (10 μg/ml) was added to 900 μl 0.2% (w/v) substrate solution. The mixture was incubated at 40°C for 30 min. For optimal pH and temperature assays and the effects of metal icons, surfactants, and chelators, the highest enzyme activity was set as 100% [25].

### Analysis of Digestion Pattern and Products

The HA was digested by HAaseD, and the reaction products were separated by thin-layer chromatography (TLC). HAaseD (100 μl, 1 U/ml) was added to HA (900 μl, 2 mg/ml) at 40°C for 0, 1, 5, 10, 15, 30, 60, and 90 min. At different time points, equal amounts of reaction products were taken by capillary tubes for TLC. N-butanol, glacial acetic acid, and water (2:2:1 [v/v/v]) were used as the developer. Aniline-diphenylamine-phosphate was used as the dye.

## Results and Discussion

### Sequence Analysis of Gene *hysA*

**Fig. 1:**
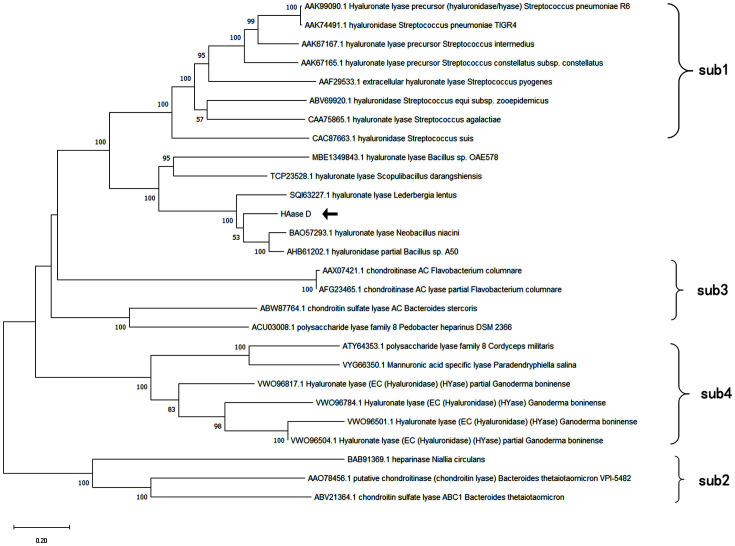
Phylogenetic tree of HAaseD and other enzymes of the PL8 family. The protein sequences were retrieved from GenBank. The phylogenetic tree was generated with the neighbor-joining method using MEGA-X. The arrow indicates the position of the HAaseD in the study.

The open reading frame (ORF) of the *hysA* was 3411 bp in length, and the deduced protein consisted of 1136 amino acid residues. The HAaseD had a relative MW of 126.2 kDa, and the pI was 5.06. The SignalP 5.0 predicted that the HAaseD contains a signal peptide (Met^1^ – Ala^30^) at the N-terminus. The mature HAaseD had an MW of 123.0 kDa, and the pI was 4.99. An NCBI CD search predicted that HAaseD has a conserved domain of glycosaminoglycan (GAG) lyase. According to the database on classification of carbohydrate-active enzymes (CAZy) [26], hyaluronate lyases belong to the polysaccharide lyase (PL) 8 family and can be categorized into four subfamilies. Based on the phylogenetic tree constructed for hyaluronate lyases of the PL8 family from the CAZy database, HAaseD was closer to the subfamily (sub) 1, but cannot be completely divided into the sub1, which indicates that it may belong to a new branch of sub1 of the PL8 family (Fig. 1). Moreover, according to BLASTP analysis, HAaseD had the highest similarity with the protein from *Bacillus* sp. A50, with the identity of 76.20%, followed by the hyaluronate lyase from *Neobacillus* niacini (75.73%). Although the hyaluronate lyases produced by the three *Bacillus* species were similar in amino acid sequence, few changes in amino acid sequence could result in significant differences in enzyme properties. In addition, *Bacillus* sp. CQMU-D was isolated from deep soil, whereas *Bacillus* sp. A50 was isolated from the air[12], and *N. niacini* was isolated from the deep-sea [13]. The hyaluronate lyase from *Bacillus* isolated from the soil is rarely reported. This study may lead to new insights into the hyaluronate lyase produced by *Bacillus*.

### Homology Modeling and Molecular Docking

**Fig. 2:**
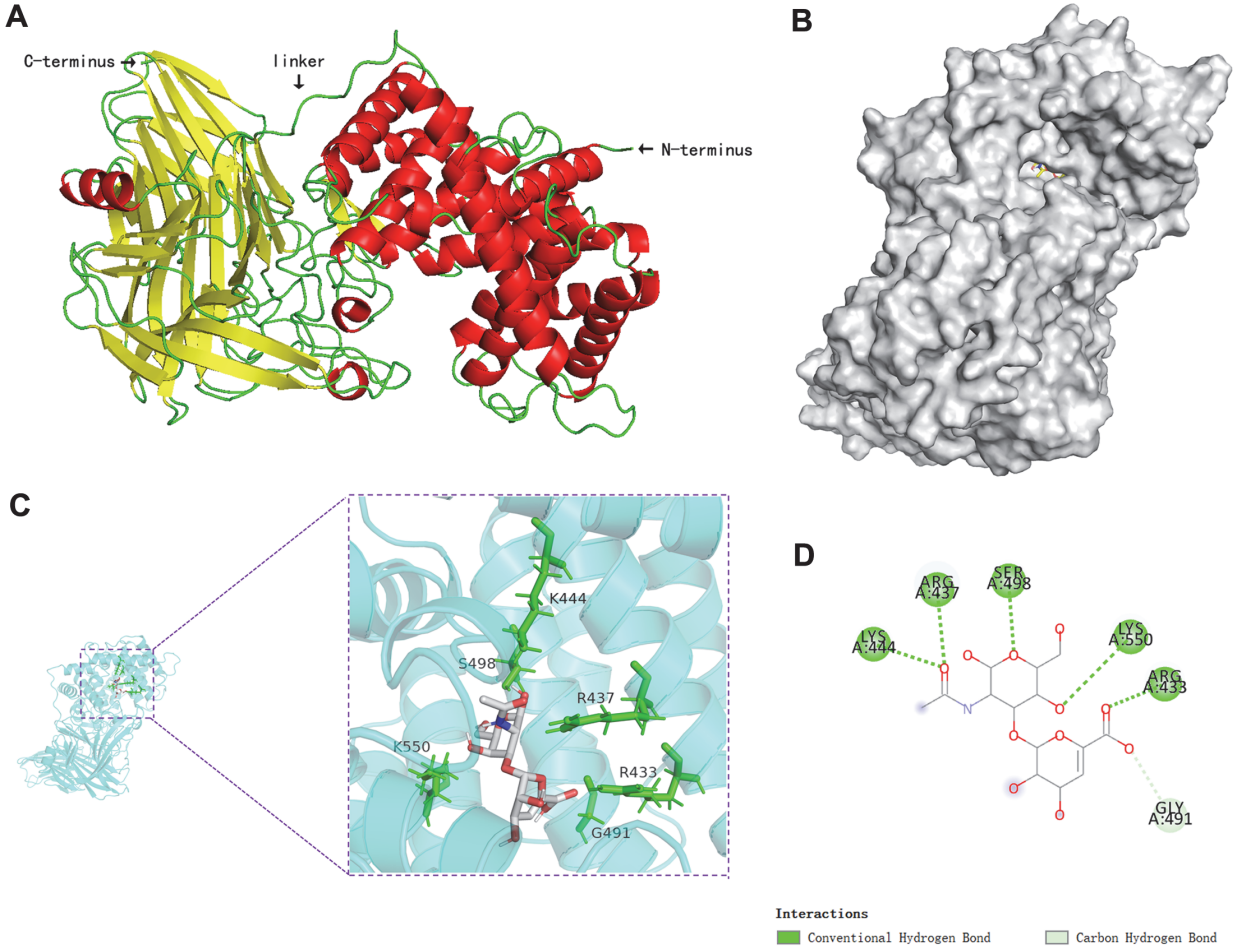
Homology modeling and molecular docking of HAaseD. (**A**) The structure of HAaseD (α-helices were shown as red; β-strands were shown as yellow; loops were shown as green). (**B**) Surface representation of HAaseD, and conserved amino acids were shown on the inside. (**C**) Cartoon representation of HAaseD. The red color indicates the presence of HA at the active site of HAaseD, while the green color indicates active residues. (**D**) Docked complex showing residues participating in HA hydrolysis.

The protein structure of HAaseD was described by PyMOL using the template accessed by SWISS-MODEL (Fig. 2). HAaseD contains two domains with a secondary structure resembling other PL8 proteins, an A domain that consists of α-helices at the N-terminus, and a B domain that contains β-sheets.

Hydrogen bonds formed by residues Arg433, Arg437, Lys444, Ser498, and Lys550 in HAaseD showed an essential role in enzyme substrate hydrolysis (Figs. 2B-2D). Small molecules interact with proteins mainly by forming hydrogen bonds and hydrophobic forces, forming hydrogen bonds with Arg433, Lys550, Ser498, Arg437 and Lys444, and hydrophobic interaction with Gly491. These sites may be the active sites of small molecules acting on the protein. Further rational design of these proteins can benefit from the prediction of 3D structures and the elucidation of interacting residues. However, it is necessary to conduct a detailed study of the interacting residues to identify key residues that may be mutated in order to improve substrate utilization.

### Production and Purification of Recombinant HAaseD

**Fig. 3:**
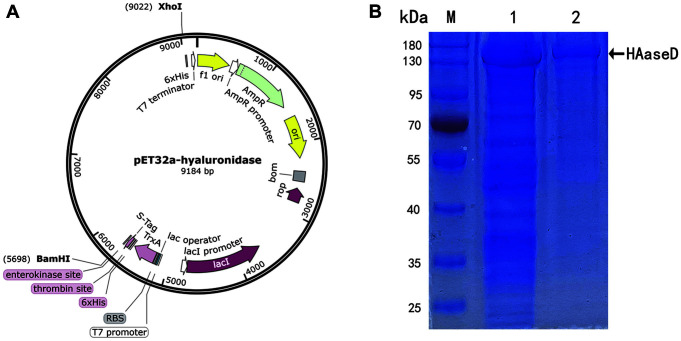
Plasmid construction and SDS-PAGE of purified HAaseD. (**A**) Schematic map of the construction of vector pET32a (+)/HAaseD. (**B**) SDS-PAGE expression analysis of HAaseD. M, protein molecular weight markers. Lane 1, total lysate proteins induced by IPTG; lane 2, SDS-PAGE fractions purified by Ni-affinity chromatography. The apparent size of the HAaseD protein is about 145 kDa.

HAaseD gene was ligated to pET32a (+) plasmid by double enzyme digestion for expression vector construction, and the gene was expressed in *E. coli* BL21 (DE3) (Fig. 3A). The recombinant protein was purified using Ni-affinity chromatography. A distinct protein band on the SDS-PAGE gel was obtained with the MW of approximately 145 kDa (Fig. 3B). A His-tagged fusion protein was produced by pET32a (+)/HAaseD, so the MW of the protein was increased, which was consistent with the expected MW.

### Characterization of HAaseD

**Fig. 4:**
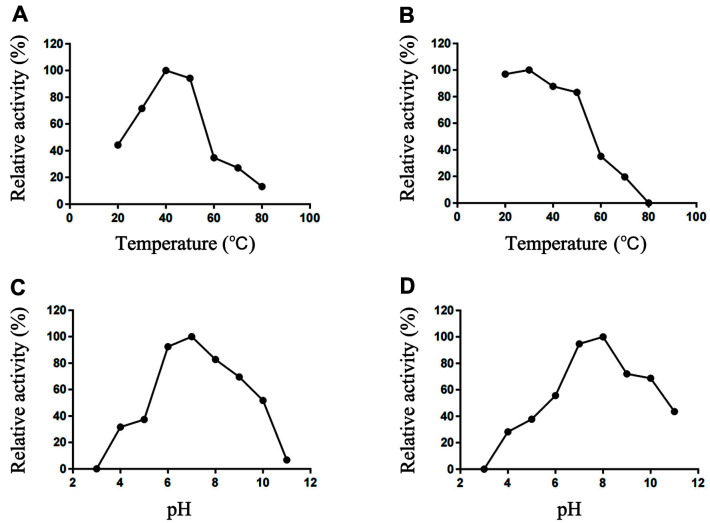
Effects of temperature and pH on the activity and stability of HAaseD. (**A**) The optimal temperature of HAaseD. (**B**) The thermostability of HAaseD. (**C**) Optimal pH of HAaseD. (**D**) The pH stability of HAaseD.

The recombinant HAaseD exhibited maximal activity at 40°C (Fig. 4A). The temperature stability of the enzyme was determined by analyzing residual activity after incubation at different temperatures for 1 h. HAaseD retained over 80% original activity after incubation at temperatures from 20 to 50°C for 1 h (Fig. 4B). However, the activity of HAaseD reduced to less than 50%, with no activity at 60 °C. The optimum temperature of HAaseD was similar to most lyases of PL8 family [27, 28], which was different from that of marine *Vibrio* LWW-9hyaluronate lyase with an optimal temperature of 30°C [29] and *Thermasporomyces composti* DSM22891 recombinant hyaluronate lyase with an optimal temperature of 70°C [25].

The optimum pH value of HAaseD determined at 40°C was 7.0 in Na_2_HPO_4_-NaH_2_PO_4_ buffer (Fig. 4C), and HAaseD activity was maintained over 60% compared to the original activity after incubation at pH ranging from 7.0-10.0 for 2 h. HAaseD also maintained more than 50% activity when incubated at pH 11.0 (Fig. 4D). Similarly, as with most PL8 family lyases, the optimum pH of HAaseD was 7.0, but it was more tolerant to alkaline conditions. Most hyaluronate lyases adapt to a pH in the range of 5.0-8.0 and are unstable under alkaline conditions, and even decline rapidly when pH exceeds 8.0 [12]. Our results showed that HAaseD remained active in alkaline conditions below pH 10.0 and remained active at pH 11.0.

**Fig. 5:**
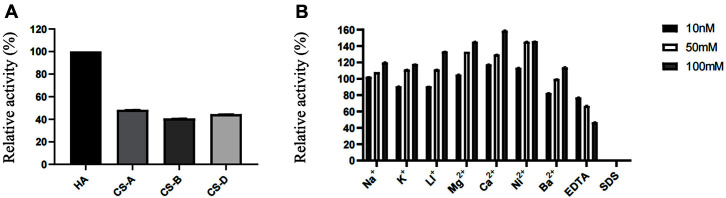
Effects of the substrate and metal ions on the activity of HAaseD. (**A**) Substrate specificity of HAaseD. HA was used as a substrate alone in all experiments except for the substrate specificity of HAaseD. (**B**) The effect of metal ions on HAaseD.

The effect of metal ions, EDTA, and SDS on the activity of HAaseD showed that HAaseD had high activity when an appropriate concentration of metal ions was added, except for Ba^2+^. Meanwhile, EDTA decreased HAaseD activity, and SDS completely inhibited enzyme activity (Fig. 5B). Owing to its metal ion tolerance characteristic, HAaseD is expected to adapt to reactions containing a variety of metal ions, and its tolerance to metal ions is higher than that of some other microbial hyaluronate lyases discovered so far. These results showed that HAaseD could degrade substrates in complex solutions with high enzymatic activity.

Under optimal reaction conditions, HAaseD had the highest catalytic efficiency for HA against different substrates (Fig. 5A). In contrast, the activity of HAaseD against various types of CS variants was relatively low, suggesting the preference for HA of HAaseD.

According to phylogenetic analysis, the GAG lyases can be divided into four subfamilies by the enzymes of the PL-8 family. Enzymes in subfamily 1 degrade only HA, those in subfamily 2 degrade CS-A, CS-C, and CS-D. Those in subfamily 3 degrade CS-A and CS-C, and with relatively weak activity toward HA [30-32]. Subfamily 4 enzymes have not yet been characterized. The results showed that HAaseD, similar to hyaluronate lyases produced by *Bacillus* sp. A50 [12], *N. niacini* [13], and *Vibrio* sp. H240 [33], had the highest degradation activity toward HA and also could degrade CS. It follows that HAaseD does not belong to any of the above subfamilies.

### Degradation Pattern and End Products of HAaseD

**Fig. 6:**
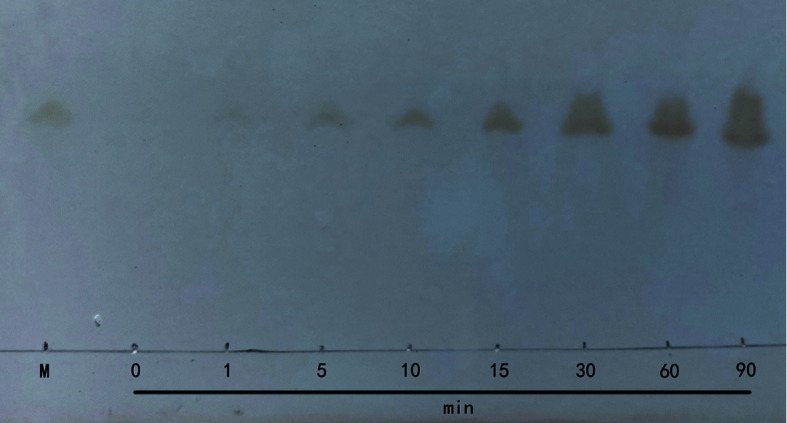
The time course of HA degradation by HAaseD was determined by TLC. M, unsaturated hyaluronan disaccharide.

To determine the degradation pattern, the cleavage products of HA degraded by HAaseD were detected using TLC. Hyaluronan oligosaccharides with low MW were present at the beginning of the reaction, and they gradually increased as the reaction progressed (Fig. 6). No other components were detected after 90 min-degradation.

According to the substrate degradation method, GAG lyase could be distinguished as endolytic and exolytic enzymes. In the same way as most PL-8 enzymes, HAaseD degrades HA into unsaturated disaccharides as a final product. Therefore, these results indicate that the prepared recombinant HAaseD is superior in the preparation of functional oligosaccharides and may have broad application prospects as tool enzymes in the future.

In this study, a novel hyaluronate lyase HAaseD originating from *Bacillus* sp. CQMU-D was characterized in vivo. The PL8 hyaluronate lyase HAaseD was confirmed to show optimum activity at 40°C and pH 7.0. The optimal substrate of HAaseD was HA, followed by CS. The endolytic type degradation pattern of HAaseD resulted in unsaturated disaccharides as the final reaction products. HAaseD retained relative stability in an alkaline environment and metal ions and was highly suitable for industrial production. The present study of HAaseD should broaden the understanding of *Bacillus* hyaluronate lyases and promotes the development and application of new hyaluronate lyases.

## References

[1] Volpí N, Schiller J, Stern R (2009). Role, metabolism, chemical modifications and applications of hyaluronan. Curr. Med. Chem..

[2] Kogan G, Soltés L, Stern R, Gemeiner P (2007). Hyaluronic acid: a natural biopolymer with a broad range of biomedical and industrial applications. Biotechnol. Lett..

[3] Fallacara A, Baldini E, Manfredini S, Vertuani S (2018). Hyaluronic acid in the third millennium. Polymers.

[4] Fraser J, Laurent T, Laurent U (1997). Hyaluronan: its nature, distribution, functions and turnover. J. Intern. Med..

[5] Kobayashi T, Chanmee T, Itano N (2020). Hyaluronan: metabolism and function. Biomolecules.

[6] Sudha P, Rose M (2014). Beneficial effects of hyaluronic acid. Adv. Food. Nutr. Res..

[7] Wang W, Wang J, Li F (2017). Hyaluronidase and chondroitinase. Adv. Exp. Med. Biol..

[8] West D, Kumar S (1989). The effect of hyaluronate and its oligosaccharides on endothelial cell proliferation and monolayer integrity. Exp. Cell. Res..

[9] Zhao Y, Qiao S, Shi S, Yao L, Hou X, Li C (2017). Modulating three-dimensional microenvironment with hyaluronan of different molecular weights alters breast cancer cell invasion behavior. ACS. Appl. Mater. Interface.

[10] Wohlrab J, Finke R, Franke W, Wohlrab A (2012). Clinical trial for safety evaluation of hyaluronidase as diffusion enhancing adjuvant for infiltration analgesia of skin with lidocaine. Dermatol. Surg..

[11] Weber G, Buhren B, Schrumpf H, Wohlrab J, Gerber P (2019). Clinical applications of hyaluronidase. Adv. Exp. Med. Biol..

[12] Guo X, Shi Y, Sheng J, Wang F (2014). A novel hyaluronidase produced by *Bacillus* sp. A50. PLoS One.

[13] Kurata A, Matsumoto M, Kobayashi T, Deguchi S, Kishimoto N (2015). Hyaluronate lyase of a deep-sea *Bacillus niacini*. Mar. Biotechnol..

[14] King S, Allen A, Maskell D, Dowson C, Whatmore A (2004). Distribution, genetic diversity, and variable expression of the gene encoding hyaluronate lyase within the *Streptococcus suis* population. J. Bacteriol..

[15] Singh S, Malhotra S, Akhtar M (2014). Characterization of hyaluronic acid specific hyaluronate lyase (HylP) from *Streptococcus pyogenes*. Biochimie.

[16] Messina L, Gavira J, Pernagallo S, Unciti-Broceta J, Sanchez Martin R, Diaz-Mochon J (2016). Identification and characterization of a bacterial hyaluronidase and its production in recombinant form. FEBS Lett..

[17] Zhu C, Zhang J, Li L, Zhang J, Jiang Y, Shen Z (2017). Purification and characterization of hyaluronate lyase from *Arthrobacter globiformis* A152. Appl. Biochem. Biotechnol..

[18] Sun X, Wang Z, Bi Y, Wang Y, Liu H (2015). Genetic and functional characterization of the hyaluronate lyase HylB and the beta-Nacetylglucosaminidase HylZ in *Streptococcus zooepidemicus*. Curr. Microbiol..

[19] Han W, Wang W, Zhao M, Sugahara K, Li F (2014). A novel eliminase from a marine bacterium that degrades hyaluronan and chondroitin sulfate. J. Biol. Chem..

[20] Maccari F, Tripodi F, Volpi N (2004). High-performance capillary electrophoresis separation of hyaluronan oligosaccharides produced by *Streptomyces hyalurolyticus* hyaluronate lyase. Carbohydr Polym..

[21] Wang L, Liu Q, Hao R, Xiong J, Li J, Guo Y (2022). Characterization of a hyaluronidase-producing *Bacillus* sp. CQMU-D isolated from soil. Curr. Microbiol..

[22] Tamura K, Stecher G, Kumar S (2021). MEGA11: Molecular evolutionary genetics analysis version 11. Mol. Biol. Evol..

[23] Khan S, Zada N, Sahinkaya M, Nigar Colak D, Ahmed S, Hasan F (2021). Cloning, expression and biochemical characterization of lignin-degrading DyP-type peroxidase from *Bacillus* sp. Strain BL5. Enzyme. Microb. Technol..

[24] Lin B, Hollingshead SK, Coligan JE, Egan ML, Pritchard DG (1994). Cloning and expression of the gene for group B streptococcal hyaluronate lyase. J. Biol. Chem..

[25] Li Y, Zhang S, Wu H, Wang X, Yu W, Han F (2020). Biochemical characterization of a thermophilic hyaluronate lyase TcHly8C from *Thermasporomyces composti* DSM22891. Int. J. Biol. Macromol..

[26] Lombard V, Golaconda Ramulu H, Drula E, Coutinho P, Henrissat B (2014). The carbohydrate-active enzymes database (CAZy) in 2013. Nucleic. Acid. Res..

[27] Sato N, Shimada M, Nakajima H, Oda H, Kimura S (1994). Cloning and expression in *Escherichia coli* of the gene encoding the *Proteus vulgaris* chondroitin ABC lyase. Appl. Microbiol. Biotechnol..

[28] Prabhakar V, Capila I, Soundararajan V, Raman R, Sasisekharan R (2009). Recombinant expression, purification, and biochemical characterization of chondroitinase ABC II from *Proteus vulgaris*. J. Biol. Chem..

[29] Wang X, Wei Z, Wu H, Li Y, Han F, Yu W (2021). Characterization of a hyaluronic acid utilization locus and identification of two hyaluronate lyases in a marine bacterium *Vibrio alginolyticus* LWW-9. Front. Microbiol.

[30] Wang W, Cai X, Han N, Han W, Sugahara K, Li F (2017). Sequencing of chondroitin sulfate oligosaccharides using a novel exolyase from a marine bacterium that degrades hyaluronan and chondroitin sulfate/dermatan sulfate. Biochem. J..

[31] Chen L, Shi C, Yin F, Wang F, Sheng J (2019). Cloning and characterization of a chondroitin AC exolyase from *Arthrobacter* sp. SD-04. Mol. Biotechnol..

[32] Sharma R, Kuche K, Thakor P, Bhavana V, Srivastava S, Mehra N (2022). Chondroitin sulfate: emerging biomaterial for biopharmaceutical purpose and tissue engineering. Carbohydr. Polym..

[33] Wang Z, Sun J, Li Y, Song G, Su H, Yu W (2022). Cloning, expression, and characterization of a glycosaminoglycan lyase from *Vibrio* sp. H240. Enzyme Microb. Technol..

